# Acceptability of Time-Limited Eating in Pediatric Weight Management

**DOI:** 10.3389/fendo.2022.811489

**Published:** 2022-04-21

**Authors:** Jared M. Tucker, Robert Siegel, Pamela J. Murray, Joan C. Han, Katherine Boyer, Nichole Reed, Taylor Allenby, Marsha Novick

**Affiliations:** ^1^ Healthy Weight Center, Helen DeVos Children's Hospital, Grand Rapids, MI, United States; ^2^ Department of Pediatrics and Human Development, Michigan State University College of Human Medicine, Grand Rapids, MI, United States; ^3^ Center for Better Health and Nutrition, Cincinnati Children's Hospital, Cincinnati, OH, United States; ^4^ Department of Pediatrics, West Virginia University School of Medicine, Morgantown, WV, United States; ^5^ Adolescent and Young Adult Medicine, Boston Children’s Hospital, Boston, MA, United States; ^6^ Department of Pediatrics, Harvard University Medical School, Boston, MA, United States; ^7^ Department of Pediatrics, Le Bonheur Children’s Hospital and University of Tennessee Health Science Center, Memphis, TN, United States; ^8^ Department of Pediatrics, Icahn School of Medicine at Mount Sinai, New York, NY, United States; ^9^ Department of General Surgery, University of Colorado School of Medicine, Aurora, CO, United States; ^10^ Pennsylvania State Medical Center, Harrisburg, PA, United States

**Keywords:** childhood obesity, intermittent fasting, nutrition, treatment, children, adolescents, time-restricted feeding (TRF)

## Abstract

**Background:**

Adherence to dietary interventions is a significant barrier in the treatment of childhood obesity. Time-limited eating (TLE) is a simple dietary approach that limits food intake to a given number of consecutive hours per day, but parental and youth acceptability of TLE in youth with obesity is unknown. This study explored the feasibility of utilizing TLE among parents and youth attending pediatric weight management (PWM).

**Methods:**

Members of COMPASS (Childhood Obesity Multi-Program Analysis and Study System) developed a survey to assess the acceptability of TLE in families attending PWM, which included patient characteristics, current diet and sleep schedules, and interests in trying TLE. The survey was administered electronically *via* REDCap or manually to parents of patients between the ages of 8-17 years old and to patients 11-17 years old attending one of five PWM practices in the COMPASS network.

**Results:**

Patients (n=213) were 13.0 ± 2.5 years old, 58% female, 52% White, 22% Black, 17% Hispanic/Latino, and 47% reported a diagnosed psychological disorder. On average, parents reported their child’s daily eating spanned 12.5 ± 1.9 hours (7:35am - 8:05pm) and included 5.6 ± 1.6 eating bouts (meals + snacks). Most parents reported being likely to try TLE ≤12 hours/d (TLE12: 66%), which was similar to the likelihood of following a nutrient-balanced diet (59%). Likelihood was lower for TLE ≤10 hours/d (TLE10: 39%) or ≤8 hours/d (TLE8: 26%) (p<0.001 for both). Interest in TLE was not consistently related to patient age, sex, or ethnicity, but was lower in patients with a psychiatric diagnosis vs. no diagnosis (TLE8: 19% vs. 32%; p=0.034). Patients of parents who reported being likely to try TLE, compared to those unlikely to try TLE, had shorter eating windows (p<0.001) and ate fewer snacks (p=0.006).

**Conclusions:**

Two-thirds of parents with children attending PWM programs report interest in TLE ≤12 hours/d regardless of demographic characteristics, but interest wanes when limiting eating to ≤10 or ≤8 hours per day. Time-limited eating appears to be a feasible option in PWM settings provided treatment options are individualized based on the interests and barriers of patients and their families.

## Introduction

Traditionally, obesity was viewed as a simple behavioral problem involving an imbalance in caloric intake and energy expenditure. However, current evidence has revealed obesity to be a multifactorial disease that involves multiple organ systems as well as behavioral, physiological, environmental, genetic and epigenetic components. One such component includes the central circadian system involved in regulating sleep/wake cycles, hormones, and metabolism. Evidence has now demonstrated a reciprocal link between the circadian system and the timing and quantity of food intake (1). For example, mutant mice with disrupted circadian systems eat outside of the typical diurnal feeding rhythm, are hyperphagic and have obesity (2). Likewise, intentional disruption of the feeding/fasting rhythms in normal-weight mice has been shown to induce obesity (3). The same phenomenon has been seen in human night-shift workers who are predisposed to obesity and increased metabolic risk (4–7).

Conversely, research in mice where food intake is limited to a particular time window each day, known as time-limited eating (TLE), reduces obesity and related metabolic disorders such as impaired glucose tolerance (8–10). A review of animal studies (11) found that, compared to ad-libitum intake, mice restricted to 8-9 hours/d of feeding experienced a 12-28% decrease in bodyweight (10, 12). A similar trend was seen in mice fed a high-fat diet within a 12 hours/d time period over 16 weeks (13), though a similar 12-week study found no effect (14). Animals that consumed a high-fat diet benefitted more from TLE than animals consuming a low-fat diet (10, 13). The TLE benefit appeared independent of calorie intake in some cases, with a similar number of calories consumed among animals on TLE and ad-libitum diets (10).

Findings from human research corroborate results from animal models, albeit with less pronounced outcomes (11). In studies involving adults who followed a 10-12 hour/d TLE plan, such as during Ramadan fasting, participants experienced 1-3% weight loss (15–21), and a concomitant decrease in blood lipids (15, 17–20), despite no difference or an increase in reported energy intake in some studies (15, 17). Similarly, studies implementing 6-8 hour/d TLE in adults with elevated weight reported weight loss ranging from 1.7-2.6% over a 3-12 week intervention period (22–24).

To date, no TLE studies have been conducted in human youth; however, TLE has recently been evaluated in adolescent mice with obesity with promising results (25) and at least one human study is currently underway (26). Children with obesity and their families often have difficulty adhering to dietary interventions that significantly alter the quantity and varieties of foods that they habitually consume (27). Time-limited eating may offer a more feasible alternative by providing simple limits to the timing of food intake, while providing fewer restrictions on the types and amounts of foods consumed. Conversely, youth may have less flexibility over mealtimes and food choices due to structured school and family schedules. The current study was designed to assess the timing of daily eating patterns for pediatric weight management patients, and to evaluate the acceptability and barriers of TLE among patients and their parents.

## Materials and Methods

### Sample

Five pediatric weight management programs from across the United States participated in the study. Programs were recruited through the Childhood Obesity Multi-Program Analysis and Study System (COMPASS), a practice-based national research network with a focus on better understanding the etiology of childhood obesity and improving its treatment through translational research. Participants for the current study were parents of patients 8 to 17 years of age who were invited to participate in the study by completing a survey while attending a treatment visit at one of the programs. If parents agreed to participate and their child was 11 to 17 years of age, the child/patient was asked to complete a similar patient version of the survey. Each program received approval from their respective Institutional Review Board (IRB) before participating.

### Measures

#### Time-Limited Eating Acceptability

All data for the current study was collected between January and November 2018 from a parent and patient/child survey administered at a PWM treatment visit. The survey used in the current study was developed by a team of clinicians and researchers specializing in childhood obesity, child psychology, and behavior analysis. The parent and child instruments contained items that assessed patient factors as they relate to TLE, including typical meal and snacking patterns, sleep habits, and acceptability of TLE, including different TLE lengths, TLE days of the week, and TLE barriers. Only the parent version of the survey included questions regarding the child’s demographic characteristics, but all other questions were identical between parent and child version. Time-of-day questions provided response options in half-hour increments, and TLE barriers were provided as a select-all-that-apply list. Acceptability was assessed using questions that asked about hypothetical TLE eating plans that differed by day of the week (school days vs. school days and weekends) and number of TLE hours per day. Assessments for TLE interest included how likely the child would be to follow four plans: 1) TLE limited eating to 12 hours per day on school days only (TLE12), 2) TLE limited to 12 hours per day on school days and weekends (TLE12week), 3) TLE limited to 10 hours per day on school days only (TLE10), and 4) TLE limited to 8 hours per day on school days only (TLE8). Response options for TLE acceptability questions used a Likert scale with 5 options ranging from “extremely unlikely” to “extremely likely”.

#### Patient Characteristics

The parent survey included questions concerning their child’s demographic and anthropometric characteristics and psychological disorders and medications for these disorders. Height and weight were parent-reported with the option for metric or imperial units, and parents were asked to use the height and weight recorded during the child’s current treatment visit, if possible. Parents were asked if their child had ever been diagnosed with the following psychological disorders: Attention Deficit/Hyperactivity Disorder (ADHD/ADD), mood disorder, such as depression or bipolar, anxiety disorder, Autism Spectrum Disorder (ASD), including Asperger’s Syndrome, and learning disabilities or delays. Any medications taken for these disorders were recorded and categorized as stimulant or non-stimulant medications. The parent survey was prefaced by a brief paragraph indicating that the completion of the survey constituted parental consent to participate and let their child participate. The patient portion of the survey was then offered to patients 11 to 17 years of age after the parent portion was completed. Both parent and patient surveys were available in English and Spanish.

### Analysis

In order to determine weight status, BMI percentile and percent of the 95^th^ BMI percentile was assessed for each child based on his/her age, height and weight using CDC growth curves (28). Then, weight status was categorized as overweight (BMI percentile: 85^th^ to <95^th^), class 1 obesity (percent of the 95^th^ BMI percentile: 100% to <120%), and severe (class 2 + class 3) obesity (percent of the 95^th^ BMI percentile: ≥120%) Due to the low number of patients defined as overweight, this group was combined with obesity for analyses. Descriptive statistics were presented as means and standard deviations for continuous variables and as frequencies and proportions for categorical data. Parent and patient characteristics were compared across programs using Pearson Chi Square as was the frequencies of survey responses across nominal groupings, such as sex. Mantel Hanzel chi square was used to evaluate linear relationships in survey responses across ordinal groups (e.g., age groups, weight status groups). Likert responses were dichotomized as “unlikely” (including “Extremely unlikely”, “Unlikely”, or “Neutral”) and “likely” (including “Likely” or “Very Likely”). Wilcoxon rank sum test (Mann-Whitney U test) was used to compare differences in the ordinal Likert responses across groups, and multiple logistic regression was used to compare group differences based on dichotomized patient responses, while adjusting for potential confounders.

## Results

A total of 222 parents agreed to review the survey’s consent page and 213 consented to participate (96%), with respondents of any single PWM program representing 8% to 44% of the total sample (median: 11%). Of participating parents, 167 had a child patient between 11-17 years of age and 159 (95%) assented to participate. Patient characteristics for each pediatric weight management program studied are displayed in **Table 1**. Mean (SD) age was 13.0 (2.5) years and the majority of patients were ≥11 years (81%), female (57%), and had severe obesity (73%). Almost half of patients had one or more parent-reported psychiatric diagnoses (44%) and almost a quarter had 2 or more diagnoses (23%), most frequently including attention deficit/hyperactivity disorder (ADHD) (23%), anxiety (19%) and mood disorders (18%). Patient characteristics often differed across programs, including mean age (p=0.024), sex (p=0.034), race/ethnicity (p<0.001), and having a diagnosed psychiatric disorder (p=0.012). Across programs, patients identifying as Black/African American ranged from 6% to 59% and those identifying as Hispanic/Latino ranged from 0 to 27%.

**Table 1:** Patient characteristics by pediatric weight management program.

		Site				
	Total *n* (%)	Program 1 *n* (%)	Program 2 *n* (%)	Program 3 *n* (%)	Program 4 *n* (%)	Program 5 *n* (%)
Total	210 (100)	56 (27)	23 (11)	92 (44)	17 (8)	22 (10)
Age Group*						
< 11 years	39 (19)	17 (30)	3 (13)	14 (15)	2 (12)	3 (14)
≥ 11 years	171 (81)	39 (70)	20 (87)	78 (85)	15 (88)	19 (86)
Sex*						
Male	89 (43)	24 (43)	7 (32)	47 (51)	2 (12)	9 (41)
Female	120 (57)	32 (57)	15 (68)	45 (49)	15 (88)	13 (59)
Race/Ethnicity*						
Black	45 (22)	16 (29)	4 (17)	11 (12)	1 (6)	13 (59)
White	109 (52)	33 (59)	14 (61)	42 (46)	14 (82)	6 (27)
Hispanic	34 (16)	5 (9)	2 (9)	25 (27)	0 (0)	2 (9)
Other	21 (10)	2 (4)	3 (13)	13 (14)	2 (12)	1 (5)
Weight Status						
Overweight^a^	5 (3)	0 (0)	0 (0)	3 (3)	0 (0)	2 (10)
Obesity^b^	47 (24)	9 (18)	5 (25)	27 (30)	4 (27)	2 (10)
Severe Obesity^c^	144 (73)	41 (82)	15 (75)	60 (67)	11 (73)	17 (81)
Psychiatric Disorder^d^*						
No	117 (56)	35 (63)	13 (57)	48 (52)	4 (24)	17 (77)
Yes	93 (44)	21 (38)	10 (43)	44 (48)	13 (76)	5 (23)

a Overweight defined as BMI percentile: 85^th^ to < 95^th^.

b Obesity (class 1) defined as percent of the 95^th^ BMI percentile: 100% to < 120%.

c Severe obesity (class 2 + class 3) defined as percent of the 95^th^ BMI percentile: ≥ 120%.

d Parents reported child psychiatric disorders defined as having been diagnosed with any of the following: attention deficit/hyperactivity disorder (ADHD/ADD), mood disorder such as depression or bipolar, anxiety disorder, autism spectrum disorder (ASD) such as Asperger’s syndrome, or learning disabilities or delays.

*Significant difference in characteristic between programs (p < 0.05).

Data were collected between January and November 2018.

### Children (8 to 10 years)

#### Eating and Sleeping Habits

Among children aged 8-10 years, parent-reported mean (± SD) sleep duration was 9.6 (0.8) hours based on an average wake time of 6:52am and an average bedtime of 9:19pm. Mean breakfast, lunch, and dinner times were 7:54am, 11:57am, and 6:24pm, respectively. In addition to meals, parents reported their child ate an average of 3.3 (2.0) snacks per day. Meals and snacks were consumed across an eating time window of 12.1 (1.3) hours. No previous dieting was reported among the majority (60%) of 8 to 10-year-olds, but those who had dieted most frequently reported calorie tracking (e.g., MyFitnessPal) (10%), My Plate (10%), or calorie restricted (7.5%) diets.

#### Time-Limited Eating Interest

Parent-reported interest in TLE was high for TLE12 (71%) but decreased for TLE10 (46%) and TLE8 (27%) and was also lower for TLE12week (59%). For all TLE options, parents of 8–10-year-olds reported similar interest as parents of older children and adolescents.

### Adolescents (11 to 17 years)

#### Eating and Sleeping Habits

Parents estimated wake times for 11 to 17-year-olds as 6:18am, on average, and bedtimes as 10:01pm (1.1) hours for a total sleep length of 8.3 (1.2) hours. Youth reported similar wake times (6:14am) but later bedtimes (10:18pm) than parents (p<0.001), resulting in a shorter mean sleep length (7.9 (1.4) hours; p<0.001).

Parent-reported mealtimes for breakfast, lunch, and dinner for the adolescents were 7:09am, 11:39am, and 6:17pm, respectively, and were similar to adolescent-reported mealtimes. Parents reported mean first daily food intake as 7:31am (due to some youth skipping breakfast) and last daily food intake as 8:06pm for a total daily eating length of 12.6 (2.0) hours. Adolescents reported similar first food intake times (7:37am) and non-significantly earlier last food intake times (7:57pm), resulting in a shorter eating length (12.2 (2.0) hours) when compared to parent-reported data (p=0.026). The number of non-meal snacks reported by parents and youth were 2.7 (1.7) and 2.6 (1.6) snacks per day, respectively.

#### Time-Limited Eating Interest

**Figure 1:**
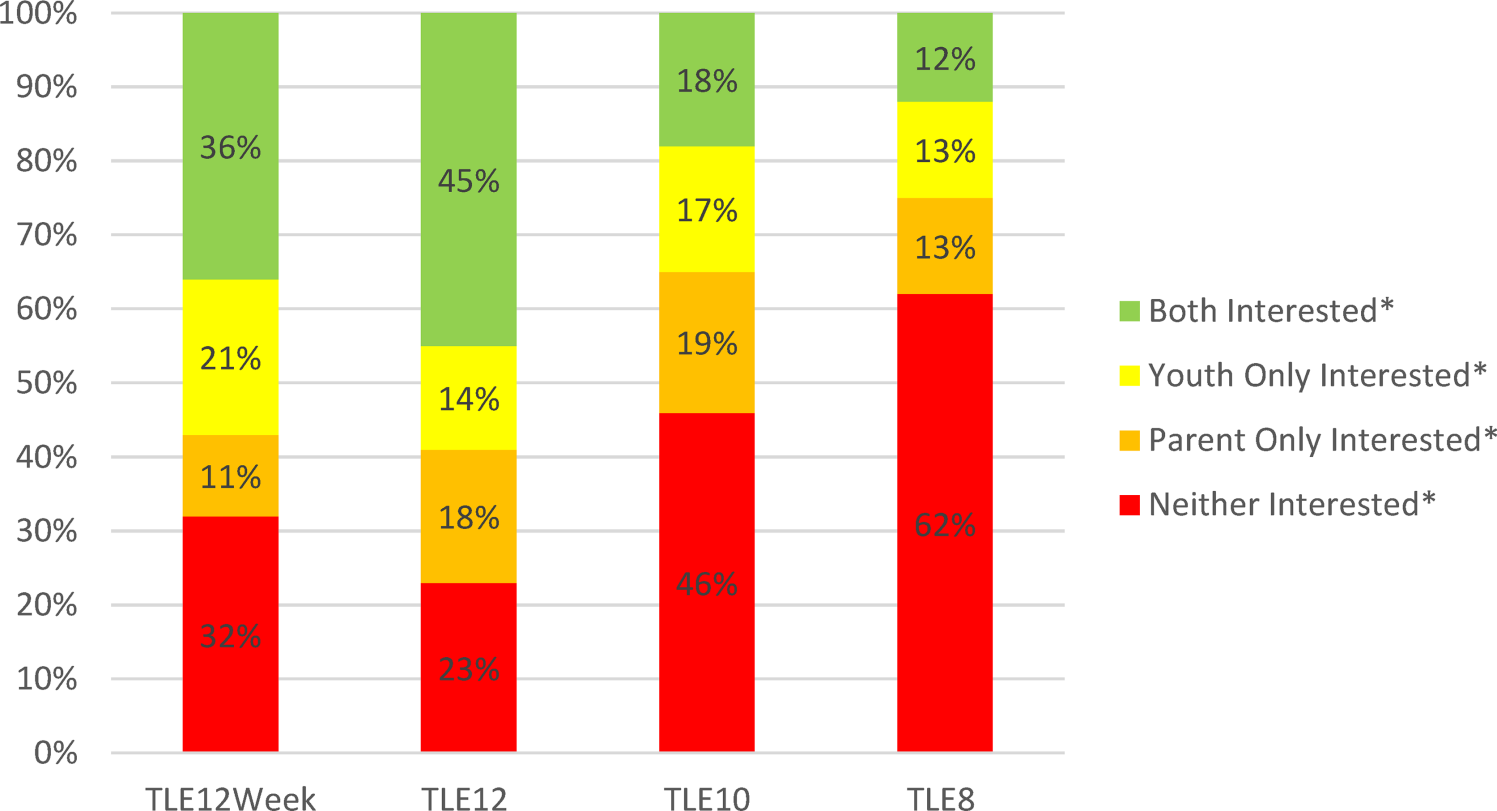
Combined Interest in Time-Limited Eating among Youth 11 to 17 years old and their Parents (n = 146). TLE12Week, Interest in time-limited eating for 12 hours per day on all days of the week; TLE12, Interest in time-limited eating for 12 hours per day on school days; TLE10, Interest in time-limited eating for 10 hours per day on school days; TLE8, Interest in time-limited eating for 8 hours per day on school days. *Interested defined as “likely” or “very likely” to follow the TLE plan in question. Data were collected between January and November 2018.

Interest in TLE did not differ between adolescents aged 11 to 13y and teens aged 14 to 17y; therefore, TLE results were combined for all youth 11 to 17y. Parent- and adolescent-reported interest in TLE is shown in **Table 2**. Similar to parents of younger children, parents of adolescents reported highest interest for TLE12 (66%), followed by TLE10 (40%) and TLE8 (26%). Adolescent interest was slightly lower across TLE12 (58%), TLE10 (35%) and TLE8 (24%) but not significantly so. When considered together, 45% of parent and adolescent dyads indicated interest in TLE12 whereas combined interest was less than half as frequent for TLE10 (18%) and TLE8 (12%) (**Figure 1**).

**Table 2:** Parent and youth interest in time-limited eating of different lengths in total and grouped by patient characteristics.

	Parent Report			Youth Report		
	TLE12^a^	TLE10^b^	TLE8^c^	TLE 12	TLE 10	TLE 8
	*n* (%)	*n* (%)	*n* (%)	*n* (%)	*n* (%)	*n* (%)
Total	134 (66)	81 (40)	52 (26)	85 (58)	51 (35)	35 (24)
Age Group						
< 11 years	27 (71)	17 (46)	10 (27)	– (–)	– (–)	– (–)
≥ 11 years	107 (64)	64 (38)	42 (25)	98 (63)	61 (39)	40 (26)
Sex						
Male	52 (61)	32 (37)	24 (28)	33 (52)	22 (35)	15 (24)
Female	81 (69)	48 (41)	28 (24)	51 (61)	29 (35)	20 (24)
Race/Ethnicity						
Black	25 (56)	22 (49)	18 (40)*	15 (54)*	9 (33)	8 (30)
White	78 (74)	41 (38)	20 (19)*	57 (71)*	34 (42)	23 (28)
Hispanic	17 (53)	12 (39)	7 (23)*	8 (33)*	7 (30)	2 (9)
Other	13 (65)	6 (29)	7 (35)*	5 (36)*	1 (7)	2 (14)
Weight Status						
Overweight/Obesity^d^	38 (75)	23 (44)	12 (23)	23 (66)	12 (35)	8 (24)
Severe Obesity^e^	89 (63)	53 (37)	36 (26)	58 (55)	37 (35)	25 (24)
Psychiatric Disorder^f^						
No	78 (70)	48 (43)	36 (33)*	50 (63)	32 (41)	24 (30)*
Yes	56 (61)	33 (35)	16 (17)*	35 (52)	19 (28)	11 (16)*

a Interest in time-limited eating for 12 hours per day on school days.

b Interest in time-limited eating for 10 hours per day on school days.

c Interest in time-limited eating for 8 hours per day on school days.

d Overweight/Obesity defined as BMI percentile: 85^th^ to percent of the 95^th^ percentile < 120%.

e Severe obesity (class 2 + class 3) defined as percent of the 95^th^ BMI percentile: ≥ 120%.

f Parents reported child psychiatric disorders defined as having been diagnosed with any of the following: attention deficit/hyperactivity disorder (ADHD/ADD), mood disorder such as depression or bipolar, anxiety disorder, autism spectrum disorder (ASD) such as Asperger’s syndrome, or learning disabilities or delays.

*Significant difference in percentages between youth grouped by a given characteristic (p< 0.05).

Data were collected between January and November 2018.

### Time-Limited Eating Associations

#### Patient Characteristics

Interest in TLE did not differ between parents and adolescents when youth were grouped by demographic characteristics. However, TLE interest did differ across some patient characteristics, including race/ethnicity and the presence of a diagnosed psychiatric disorder (**Table 2**). For parents, TLE8 interest differed by their child’s race/ethnicity (p=0.036) with highest interest among parents of Black/African American youth (40%) and lowest interest among parents of White youth (18%). For adolescents, interest in TLE12 differed across race/ethnicity (p=0.002) with the highest interest among White youth (71%) and lowest interest among Hispanic youth (33%). Interest in TLE8 was also higher among those without a psychiatric diagnosis when compared to those with one or more psychiatric diagnoses for both parents (33% vs. 17%; p=0.012) and adolescents (24% vs. 11%; p=0.049).

#### Eating and Sleeping Habits

Associations between parent interest in TLE and their child’s eating habits are presented in **Table 3**. Those interested in TLE12 reported fewer snacks (p=0.006) and fewer total bouts of eating (meals + snacks) (p=0.013) than those not interested in TLE12. Parents interested in TLE10 reported no difference in eating bouts or snacks than those not interested, but did report shorter daily eating durations (p<0.001) among their children due to earlier consumption of their last meal or snack (p=0.023). Shorter daily eating duration was also associated with parent interest in TLE8 (p=0.020). Interest in TLE was not associated with bedtimes, wake times, or sleep durations.

**Table 3:** Eating and sleeping habits grouped by interest in time-limited eating of different lengths among parents of 8-17 year-old patients.

	*n*	First Food Intake (Time)^a^ Mean (SD)	Last Food Intake (Time) Mean (SD)	Eating Duration (Hours) Mean (SD)	Snacks (# per day) Mean (SD)	Wake time (Time) Mean (SD)	Bedtime (Time) Mean (SD)
**Total**	191	7.6 (1.5)	20.1 (1.3)	12.5 (1.9)	2.8 (1.6)	6.4 (0.9)	21.9 (1.1)
**TLE12week**^b^	191						
**No interest**	79	7.6 (1.5)	20.3 (1.5)	12.7 (2.0)	3.1* (2.0)	6.4 (0.7)	22.0 (1.2)
**Interest**	112	7.6 (1.6)	20.0 (1.1)	12.3 (1.7)	2.5* (1.3)	6.4 (0.9)	21.8 (1.0)
**TLE12**^c^	190						
**No interest**	65	7.6 (1.4)	20.3 (1.4)	12.7 (1.9)	3.3* (2.0)	6.5 (0.8)	21.9 (1.1)
**Interest**	125	7.6 (1.6)	20.0 (1.2)	12.3 (1.8)	2.5* (1.4)	6.4 (0.9)	21.9 (1.0)
**TLE10**^d^	191						
**No interest**	117	7.4 (1.4)	20.3* (1.3)	12.8* (1.8)	2.9 (1.6)	6.4 (0.8)	21.9 (1.1)
**Interest**	74	7.8 (1.7)	19.8* (1.2)	11.9* (1.8)	2.6 (1.7)	6.4 (0.9)	21.9 (1.1)
**TLE8**^e^	189						
**No interest**	140	7.5 (1.5)	20.2 (1.3)	12.7* (1.9)	2.9 (1.7)	6.4 (0.8)	21.9 (1.1)
**Interest**	49	7.9 (1.8)	19.9 (1.1)	11.9* (1.8)	2.5 (1.5)	6.5 (1.0)	21.8 (1.1)

a Time calculated as the number of hours after 12:00am.

b Interest in time-limited eating for 12 hours per day on school days.

c Interest in time-limited eating for 12 hours per day on school days.

d Interest in time-limited eating for 10 hours per day on school days.

e Interest in time-limited eating for 8 hours per day on school days.

*Significant difference in means between TLE interest vs. no interest (p < 0.05).

Data were collected between January and November 2018.

When testing for linear associations between TLE and daily snack intake, parent interest in TLE12 was negatively associated with the number of snacks their child consumed daily (p=0.018) such that 79% of parents reporting 0-1 snacks per day were interested in TLE12, but interest dropped among parents reporting 2-3 snacks (69%), 4-5 snacks (51%), and 6+ snacks per day (55%). Parent interest in TLE10 and TLE8 showed similar, though non-significant, inverse trends (p<0.1) with snacking. For adolescents, interest in TLE was not associated with the number of snacks they reported.

### Barriers to TLE

**Figure 2:**
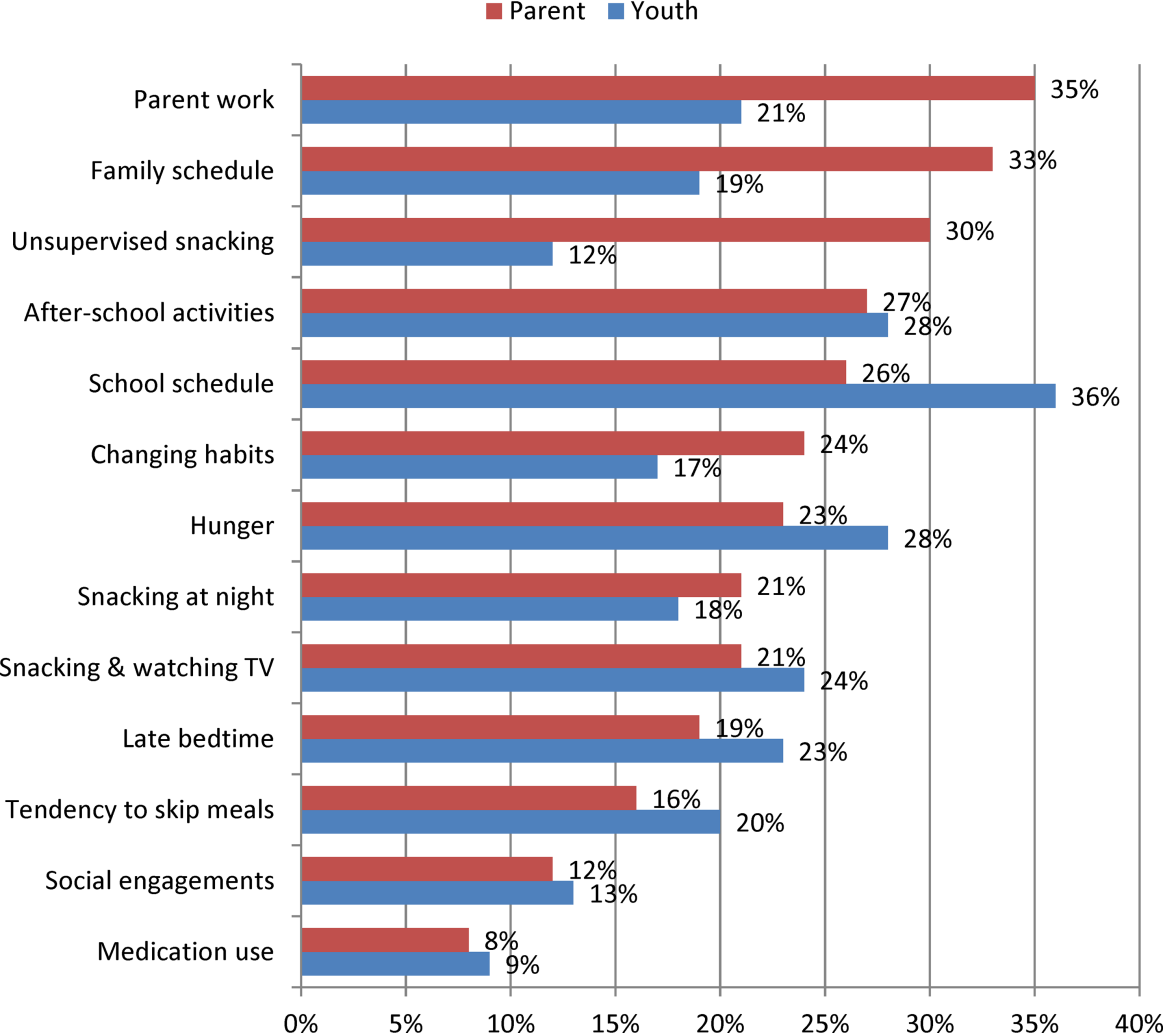
Frequency of Perceived Barriers to Time-Limited Eating among Parents of Youth 8 to 17 years old (n = 213) and among Youth 11 to 17 years old (n = 159). Data were collected between January and November 2018.

Perceived barriers to TLE for parents and youth are shown in **Figure 2**. For parents, the most frequently reported anticipated barriers to TLE included scheduling obstacles, such as parent work (35%), family schedule (33%), school schedule (26%) and after-school activities (27%), though unsupervised snacking was also a common concern (30%). Youth also frequently anticipated some scheduling barriers, including school schedule (36%) and after-school activities (28%), but also reported hunger (28%), snacking while watching TV (24%) and late bedtimes (23%) as commonly anticipated difficulties.

Those interested in TLE perceived some barriers at different frequencies than those not interested in TLE. *Snacking while watching TV* was perceived as a barrier less frequently for those interested in TLE12 (15% vs. 34%; p=0.001) and TLE10 (10% vs. 29%; p=0.001). Similarly, *family schedule* was seen as a barrier less frequently for those interested in TLE10 (26% vs. 40%; p=0.045) and TLE8 (19% vs. 40%; p=0.007) as was *parent work* for TLE8 (23% vs. 40%; p=0.025). Among adolescents, interest in TLE12 was associated with a lower frequency of the following barriers: late bedtime (15% vs. 32%; p=0.016), snacking at night (11% vs. 27%; p=0.028), snacking while watching TV (16% vs. 33%; p=0.022), and snacking without permission (5% vs. 19%; p=0.013). *School schedule* was viewed as a barrier less frequently for those interested in TLE10 (28% vs. 45%; p=0.035), while no differences in barrier frequency were seen for TLE8.

## Discussion

This acceptability study included 213 patients ages 8-17 years old and their caretakers participating in five tertiary pediatric weight management programs across the United States to determine the interest in a TLE program for their child. The results show that TLE appears to be an acceptable treatment option among some pediatric patients with obesity who are seen in a pediatric weight management setting, provided that treatment options are individualized and based on the interests and barriers of patients and their families.

In this study, two-thirds of parents and children attending these programs reported interest in TLE12, regardless of demographic characteristics. This finding was similar to the likelihood of following a nutrient-balanced diet (59%). High acceptability of TLE12 is not surprising considering parents of 11-17 year-olds reported that their child’s eating spanned 12.6 hours, on average, and parents of 8-10 year-olds reported an eating time window spanning 12.1 hours. Unlike TLE10 and TLE8, interest TLE12 did not differ by the typical daily length of eating in youth, suggesting this interval may not have felt restrictive based on current habits. Therefore, despite the increased feasibility of a 12-hour eating window when compared to shorter eating intervals, TLE12 may not meaningfully influence eating timing in youth seeking obesity treatment. Therefore, determining the hours of eating for a TLE plan in youth would need to be strategic, previously agreed upon, and may need to be reduced in the middle of a TLE plan to have beneficial health outcomes.

Our findings showed a substantial decrease in interest in TLE in both parents and children when questioned about TLE10 and TLE8. This is not surprising considering these diets are more restrictive compared to current eating habits. We hypothesized that interest in shorter TLE plans would be higher among older youth when compared to younger children, since adolescents may be better able to manage their hunger; however, TLE interest did not differ across age groups for any time interval. Perhaps the increased maturation and potential dietary restraint of older youth was offset by their later bedtimes and longer daily eating windows. This is evidenced by the fact that, regardless of patient age, parents who reported being likely to try TLE10 and TLE8 had eating windows that were almost an hour shorter than those unlikely to try these plans. In addition, parents may perceive less control over the food choices of older adolescents, which may reduce their interest in attempting a TLE plan.

Though no data are available in youth, adult TLE research supports relatively high levels of acceptability and adherence. In a recent 21-day randomized controlled trial (22), women with obesity assigned to a hypoenergetic diet with or without TLE12 did not differ in self-reported hunger or difficulty adhering to the protocol, and there was no loss to follow up in the TLE12 group. In a study implementing TLE10 and using a validated app to track dietary intake (20), the eating window of 19 adults with metabolic syndrome was reduced from 15.1h to 10.8 h, and participants ate outside their eating window on only 7.1% of days, indicating high adherence to the protocol. Two short-term interventions evaluating TLE8 reported 84% adherence (i.e., percent fasting 14-18 h per day during weeks 2-4) (24) and 80% adherence (i.e., percent of days eating between 10am to 6pm during 12 weeks) (23). Thus, compliance appears to be a strong suit of TLE interventions among adults, at least in the short term. However, youth may experience barriers to TLE that adults do not.

According to our findings, both parents and youth often foresee schedule-related barriers as well as behavior-based challenges to adopting a TLE plan. When asked about anticipated barriers to TLE, the top two concerns described by parents were the parent’s work schedule (35%) and the family schedule (33%), while youth most frequently reported school schedule (36%) and after-school schedule (28%) as anticipated barriers. However, the third most frequent barrier was behavior-based for both parents (unsupervised snacking: 30%) and youth (hunger: 28%). Therefore, when determining whether TLE would be an appropriate treatment plan for a pediatric patient with obesity, the family schedule, including work, school and after-school arrangements, including accommodation of different eating schedules, should be a part of the conversation, as should potential strategies to tailor behaviors and environments to bolster TLE compliance. Such strategies may include eliminating tempting foods from the home, meal planning, minimizing boredom and providing structure during fasting periods, and adjusting sleep schedules to avoid late-night hunger and snacking.

In our study, interest in TLE was not consistently related to the patient’s age, sex or weight status, but was lower in participants with a psychiatric diagnosis compared to participants without a psychiatric diagnosis for both TLE10 and TLE8. Given the high association between attending a pediatric weight management program and having a psychiatric comorbidity (47% in this study though somewhat lower in others) (29), a patient’s psychological history should be determined and considered before using TLE as a treatment option.

In addition, the amount of patient snacking per day was associated with interest in TLE. Parents reported an average of 2.8 snacks daily with approximately one-quarter of the children eating four or more snacks per day. We found that patients of parents who reported being likely to try TLE ate fewer snacks than youth of parents unlikely to try TLE such that 79% of parents reporting interest in TLE12 if their child ate 0-1 snacks per day compared to 69% for children eating 2-3 snacks per day and 51% for children eating 4-5 snacks per day. Therefore, lower rates of snacking, was a sign of improved interest in a TLE plan; however, families with higher snacking levels may benefit more from TLE. Thus, motivation to reduce snacking intake may be necessary for successful implementation of a TLE plan among youth with frequent bouts of eating.

For those children and parents who are interested in TLE, successful implementation may provide health gains and quality of life benefits. For instance, adolescents who socialize and snack with friends would not have to disclose that they are “on a diet” and would be able to adhere to a TLE plan without social stigma. For younger children, creating set times and expectations of a meal and snack schedule may allow for easier implementation to reduce calorie intake and adherence to reducing snacks later in the day. Lastly, mealtime eating and snacking often changes during the summertime, causing an increased rate of BMI gain for children (30, 31). Based on the “structured day hypothesis”, creating an eating schedule through TLE may help assuage excess BMI gains during the summer by providing a more consistent schedule similar to the structure of school weekdays (32).

Strengths of this study include the assessment of both parent and adolescent perspectives on TLE feasibility among families attending pediatric weight management. This research topic is unique, given the limited information in the current literature as to whether children and adolescents would be interested in attempting a TLE program. In addition, this research study includes a cohort of 213 patients from 5 different pediatric weight management programs nationwide and includes patients with diverse demographic characteristics which provides broad generalizability for this treatment-seeking population. A limitation of this study includes self-reported nature of youths’ daily habits such as sleep, meal timing and snacking; however, providing both parent and youth responses allows for greater confidence in the validity of these estimates. Also, parent finances and work schedules likely influence perceived barriers to TLE, yet we did not collect these data as we were concerned the sensitivity of such questions may have reduced response rates. Another limitation is that patient behaviors likely vary depending on the time of the year due to seasonal factors like weather and school schedules. While time of year was not accounted for in this study, the majority of data were collected during the academic school year (spring and fall) in order to provide comparable results.

Based on our findings, TLE would likely be well-received by many parents and youth enrolled in pediatric weight management. Future research is needed to implement a TLE protocol to youth participating in clinically supervised obesity treatment. Optimally, a TLE intervention could be compared to patients not undergoing TLE (e.g., a reduced calorie or nutrition-balanced plan) or perhaps even a different TLE duration (e.g., TLE12 vs TLE8). Patients who snack frequently, snack without parental permission, have parents or families with busy work and after-school schedules, or have a psychiatric diagnosis should be invited to participate with caution as these groups may perceive more barriers to TLE. Ultimately, a “shared decision making” approach should be used to guide obesity treatment for patients and their families (33, 34). This approach is patient-centered such that clinicians focus on the needs and preferences of the patient/family and share control of the consultation (35). Shared decision making is a best practice when more than one treatment option may be appropriate, which is typically true of nutrition-based approaches in pediatric weight management. Using such an approach, our findings suggest TLE could serve as a feasible nutritional intervention that many pediatric weight management families may be interested in pursuing.

## Data Availability Statement

The raw data supporting the conclusions of this article will be made available by the authors, without undue reservation.

## Ethics Statement

The studies involving human participants were reviewed and approved by Spectrum Health Human Research Protection Program, Cincinnati Children’s Hospital Medical Center Institutional Review Board, West Virginia University Office of Research Integrity and Compliance, Penn State Human Research Protection Program, The University of Tennessee Health Science Center Institutional Review Board. Written informed consent to participate in this study was provided by the participants’ legal guardian/next of kin.

## Author Contributions

JT, MN, PM, JH, and RS contributed to the conception and design of the study. All authors contributed to the methodology and data collection. JT organized the database and performed the statistical analysis. JT and MN wrote the first draft of the manuscript. All authors contributed to manuscript revision, read and approved the final version.

## Conflict of Interest

The authors declare that the research was conducted in the absence of any commercial or financial relationships that could be construed as a potential conflict of interest.

## Publisher’s Note

All claims expressed in this article are solely those of the authors and do not necessarily represent those of their affiliated organizations, or those of the publisher, the editors and the reviewers. Any product that may be evaluated in this article, or claim that may be made by its manufacturer, is not guaranteed or endorsed by the publisher.
